# Downregulation of miR-221 Inhibits Cell Migration and Invasion through Targeting Methyl-CpG Binding Domain Protein 2 in Human Oral Squamous Cell Carcinoma Cells

**DOI:** 10.1155/2015/751672

**Published:** 2015-12-16

**Authors:** Shuqi He, Renfa Lai, Dan Chen, Wangxiang Yan, Zhaoqiang Zhang, Zhiguo Liu, Xueqiang Ding, Yu Chen

**Affiliations:** ^1^The Medical Centre of Stomatology, The First Affiliated Hospital of Jinan University, Guangzhou 510630, China; ^2^Department of Oral & Maxillofacial Surgery, First Affiliated Hospital of Sun Yat-Sen University, Guangzhou 510080, China; ^3^Department of Stomatology, Sixth Affiliated Hospital of Sun Yat-Sen University, Guangzhou 510655, China; ^4^Department of Oral & Maxillofacial Surgery, Guanghua School of Stomatology, Guangdong Provincial Key Laboratory of Stomatology, Sun Yat-Sen University, Guangzhou 510055, China

## Abstract

Oral squamous cell carcinoma (OSCC), the most frequent of all oral cancers, is a type of highly malignant tumors with a high capacity to invade locally and form distant metastases. An increasing number of studies have shown that microRNAs (miRNAs) play an important role in regulating cancer metastasis and invasion. In the present study, we detected the expression of miR-221 in two highly metastatic OSCC cell lines and two OSCC cell lines that are less metastatic using quantitative real-time PCR analysis (qRT-PCR). The qRT-PCR results indicate that miR-221 is upregulated in highly metastatic OSCC cell lines. Then, miR-221 expression was knocked down by transfection with miR-221 inhibitor, and UM1 cell migration and invasion were assessed using transwell migration and invasion assays. The results indicate that inhibition of miR-221 suppressed migration and invasion of UM1 cells. Furthermore, methyl-CpG binding domain protein 2 (MBD2) was identified as a direct target gene of miR-221. Additionally, MBD2 silencing could partly reverse the effect of miR-221 on cell migration and invasion. In conclusion, downregulation of miR-221 inhibits cell migration and invasion at least partially through targeting MBD2 in the human OSCC cell line UM1.

## 1. Introduction

Oral cancer, a type of head and neck cancer, is any cancerous tissue growth located in the oral cavity. Oral cancer has been identified as a significant worldwide public health threat because its treatment often produces dysfunction and distortions in speech, mastication and swallowing, dental health, and even the ability to interact socially [1]. Oral squamous cell carcinoma (OSCC) represents the most frequent of all oral cancers, and more than 90% of oral cancers are diagnosed as OSCC [2, 3]. Although local OSCC can be effectively controlled by surgical excision and radiotherapy, metastasis to the lymph nodes and distant organs significantly decreases survival rate [4]. As OSCC is a type of highly malignant tumor with a large capacity to invade locally and metastasize, an approach that decreases invasion and metastasis may facilitate the development of an effective adjuvant therapy [1]. The invasion of tumor cells is a complex, multistage process. It is therefore necessary to identify critical targets in OSCC metastasis such that effective treatments can be developed.

MicroRNAs (miRNAs) are small noncoding RNA molecules (containing approximately 22 nucleotides) that function in RNA silencing and posttranscriptional regulation of gene expression through binding to the 3′-untranslated region (UTR) of target genes [5, 6]. Previous studies have revealed that miRNAs play an important role in regulating cancer metastasis and invasion [7–10]. miR-221 belongs to the miR-221/222 clusters, which are encoded in tandem on the X chromosome in human, mouse, and rat and are highly conserved in vertebrates [11]. Moreover, they have the same seed sequence. An increasing number of studies have demonstrated that miR-221 can function as a potential oncogene or a tumor suppressor gene, depending on the target genes [11]. The function of miR-221 in cancer cell metastasis and invasion has been examined in multiple types of cancers, including gliomas, colon cancer, and renal cell carcinoma [12–14]. These studies demonstrated that miR-221 acts as an oncogene in these cancers. In addition, previous studies have reported the function of miR-221 in OSCC. In the study of Yang and coworkers, the expression level of miR-221 was highly correlated with cell growth in OSCC [15].

The exact function of miR-221 in cancer metastasis and invasion of OSCC remains unclear. In this study, we focused on demonstrating the function of miR-221 in OSCC metastasis and invasion, and we identified the target of miR-221 related to metastasis and invasion. The present study revealed that miR-221 is upregulated in highly metastatic OSCC cell lines and that downregulation of miR-221 inhibits cell migration and invasion partly through targeting methyl-CpG binding domain protein 2 (MBD2).

## 2. Materials and Methods

### 2.1. Cell Lines and Culture

The OSCC lines CAL-27, Tca8113, UM1, and UM2 [16] were cultured in Dulbecco's modified eagle medium (DMEM) supplemented with 10% fetal bovine serum (Gibco), penicillin (100 U/mL), and streptomycin (100 *μ*g/mL). Cells were maintained at 37°C in a humidified incubator with 5% CO_2_ and were passaged upon reaching 90–95% confluence.

### 2.2. miRNA Mimics and siRNA Transfection

A negative control (miR-NC), miR-221 mimic, and miR-221 inhibitor were purchased from Jima Biotech (Suzhou, China). miR-221 inhibitor is chemically modified antisense oligonucleotide, which can compete against endogenous miRNAs in RNA-induced silencing complex incorporation. A small interfering RNA against MBD2 (si-MBD2) and a negative control (si-NC) were purchased from Santa Cruz Biotechnology (Santa Cruz, CA, USA). Cells were plated at 50% confluence and transfected with 300 nM miR-221 mimic or 10 *μ*M siRNA using Lipofectamine RNAiMAX Transfection Reagent (Invitrogen, CA, USA), according to the manufacturer's protocol. Cells were harvested at 24 or 48 h after transfection for further analysis.

### 2.3. RNA Extraction and Quantitative Real-Time PCR Analysis (qRT-PCR)

Total RNA was extracted from harvested cells using Trizol reagent (Invitrogen, CA, USA). To analyze miR-221 expression, reverse transcription PCR was performed using specific stem-loop reverse transcription primers, miRNA first strand synthesis was performed using a First Strand Synthesis Kit (Takara, Dalian, China), and qRT-PCR was performed using a Mir-X miRNA qRT-PCR SYBR Kit (Takara, Dalian, China) on an Applied Biosystems 7500 system (Applied Biosystems, Warrington, UK). U6 was used as an internal control.

To quantify mRNA levels of MBD2, reverse transcription PCR was performed using PrimeScript RT Reagent Kit with cDNA Eraser (Takara, Dalian, China), and qRT-PCR was performed using SYBR Premix Ex Taq (Takara, Dalian, China). GAPDH was used as an internal control. The primer sequences used in qRT-PCR are shown in Table 1. Gene expression was measured in triplicate, quantified using the 2^−ΔΔCT^ method, and normalized to a control.

### 2.4. Transwell Migration and Invasion Assays

Cell migration and invasion were assessed using a transwell assay. For migration, UM1 cells were harvested and 5 × 10^4^ cells in 200 *μ*L of 0.1% serum medium were placed in the upper chamber of an insert (pore size, 8 *μ*m) (Becton Dickinson Labware). The lower chamber was filled with 10% fetal bovine serum medium (600 *μ*L). For invasion, the same density of cells was placed into the upper chamber precoated with Matrigel (BD Biosciences, Bedford, MA, USA). After 24 h incubation and removal of the cells on the upper chamber of the filter with a cotton swab, the cells on the underside were fixed with 4% paraformaldehyde, stained with 0.1% crystal violet in 20% ethanol, and counted in five randomly selected fields using a phase contrast microscope. Migrating cells were monitored by photographing at 200x magnification with a LEICA microscope (Darmstadt, Germany) in five independent fields for each well. The assays were performed in triplicate.

### 2.5. Wound Healing Assay

For studying cell migration in a scratch wound assay, UM1 cells were seeded in 6-well plates and artificial wounds were inflicted to the cell layer by scratching with sterile 200 *μ*L pipette tips. For each condition, three scratches were inflicted in three independent wells of a 6-well plate. From each of these scratches, eight images were taken for a total of 24 images per condition and time point. Images were performed by phase contrast microscopy (Leica, Darmstadt, Germany) immediately after wounding and after 24 h. The migrated area of cells into the wound was quantified with Image Pro Plus 6.0 software.

### 2.6. Western Blotting

Each group of UM1 cells was lysed using RIPA buffer (Beyotime Biotechnology, Nantong, China). The total protein concentration was determined using a BCA Protein Assay kit (Beyotime Biotechnology, Nantong, China). Equal amounts of total protein were loaded in tracks, separated on 8% SDS polyacrylamide gels, and transferred to PVDF membranes (Pall, New York, NY, USA). Membranes were blocked for 1 h at room temperature with 5% milk in TBS containing 0.05% Tween-20 (TBST), incubated for 1 h with rabbit anti-human MBD2 monoclonal antibody (1 : 5000, ab109260, Abcam, Cambridge, MA, USA) or rabbit anti-human beta actin monoclonal antibody (1 : 2000, ab119716, Abcam, Cambridge, MA, USA), and washed three times with TBST. Membranes were incubated with horseradish peroxidase-conjugated goat anti-rabbit IgG H&L secondary antibody (1 : 10000, ab97080, Abcam, Cambridge, MA, USA) for 40 min and washed three times with TBST, and proteins were visualized using ECL (Thermo Scientific Pierce ECL Plus).

### 2.7. Reporter Vector Construction and Luciferase Reporter Assay

The miRNA target prediction software programs Targetscan (http://www.targetscan.org) and miRanda (http://www.microrna.org/microrna/home.do) were used to predict the targets of miR-221. The full-length wild-type 3′-UTR of MBD2 (NM_003927) and mutant 3′-UTR of MBD2 were amplified and cloned into the psi-CHECK-2 vector (Promega, Madison, WI, USA). The primer sequences used in the reporter vector construction are shown in Table 2. All inserts and plasmids were verified by DNA sequencing. UM1 cells, plated on 24-well plates, were cotransfected with 100 ng plasmid and 200 nmol/L miR-221 mimic or miR-NC. Cell lysates were harvested 48 h after transfection, and firefly and* Renilla* luciferase activities were measured by the Dual-Luciferase Reporter Assay System (Promega, Madison, WI, USA), according to the manufacturer's instructions. Three independent experiments were performed.

### 2.8. Statistical Analysis

All statistical analyses were performed using SPSS 19.0 software (IBM, Chicago, IL, USA). Results are represented as means ± standard deviation (SD). Student's *t*-test was used to compare means from different groups; *P* values < 0.05 were regarded as statistically significant.

## 3. Results

### 3.1. miR-221 Is Upregulated in Highly Metastatic OSCC Cell Lines

**Figure 1:**
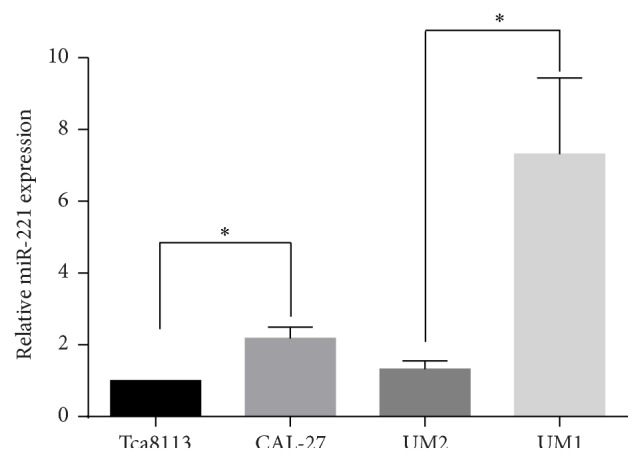
miR-221 is upregulated in highly metastatic OSCC cell lines. The expression level of miR-221 in two highly metastatic OSCC cell lines (CAL-27 and UM1) and two less metastatic OSCC cell lines (Tca8113 and UM2) was detected using qRT-PCR. The results are presented as means ± SD. ^*∗*^ *P* < 0.05.

To investigate the role of miR-221 in regulating OSCC cell migration and invasion, we detected the miR-221 expression level in two highly metastatic OSCC cell lines (CAL-27 [17] and UM1 [16]) and two less metastatic OSCC cell lines (Tca8113 [18] and UM2 [16]) using qRT-PCR. The results demonstrated that the expression level of miR-221 was increased in the highly metastatic OSCC cell lines compared to the less metastatic cell lines (Figure 1). The expression of miR-221 was the highest in the OSCC cell line UM1. Based on these results, we chose the UM1 cell line for further analyses.

### 3.2. miR-221 Inhibitor Could Effectively Suppress miR-221 Expression Level

**Figure 2:**
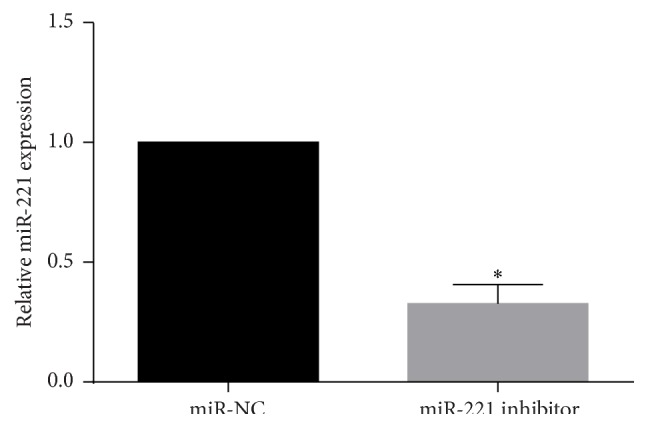
The expression level of miR-221 after miR-221 inhibitor transfection for 48 h detected using qRT-PCR. Results are presented as means ± SD. ^*∗*^ *P* < 0.05.

Since the expression level of miR-221 is increased in the highly metastatic OSCC cell line UM1, we transfected a miR-221 inhibitor into UM1 cells. Then, cells were harvested for qRT-PCR. The results indicate that the miR-221 inhibitor could effectively suppress miR-221 expression (Figure 2).

### 3.3. Inhibition of miR-221 Suppressed Migration and Invasion of UM1 Cells

**Figure 3:**
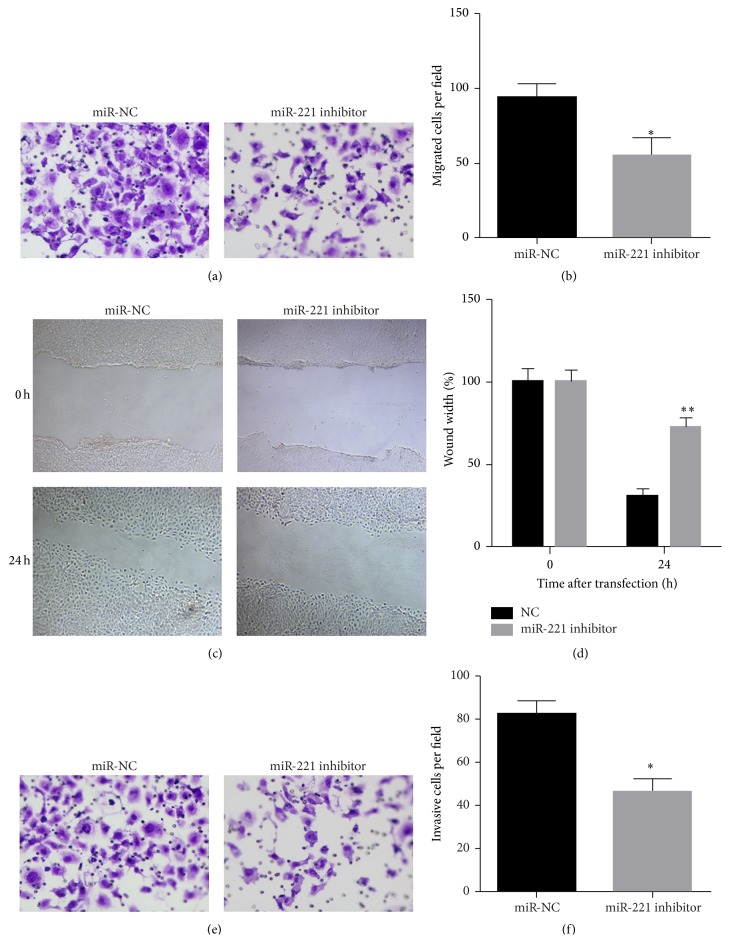
Inhibition of miR-221 suppressed migration and invasion of UM1 cells. (a) Representative images of UM1 cell migration are shown. The migration of UM1 cells was measured using a transwell assay at 48 h after transfection with miR-221 inhibitor or miR-NC. (b) The average number of migrating cells per field for the indicated experimental groups is shown. (c) Representative images of UM1 migration cells analyzed by wound healing assays. Images show migration of cells after 0 h and 24 h. (d) Quantification of migrated UM1 cells analyzed by wound healing assays. The migration of UM1 cells transfected with miR-NC set to 100%. (e) Representative images of UM1 cell invasion are shown. The invasion of UM1 cells was measured using a Matrigel invasion assay at 48 h after transfection with miR-221 inhibitor or miR-NC. (f) The average number of invading cells per field for the indicated experimental groups is shown. Data are presented as means ± SD. ^*∗*^ *P* < 0.05; ^*∗∗*^ *P* < 0.01.

To demonstrate the role of miR-221 in regulating UM1 cell migration and invasion, transwell and wound healing assays were performed after miR-221 inhibitor or miR-NC transfection. For transwell migration assays, the number of cells that passed through the membrane onto the lower chamber was significantly less in the miR-221 inhibitor transfected cells than in miR-NC transfected cells (Figure 3(a)). The number of migrating cells after transfection with miR-NC or the miR-221 inhibitor was 94 ± 9 and 55 ± 12, respectively (*P* < 0.05) (Figure 3(b)). In addition, the wound healing assay showed that the migratory ability of UM1 cells transfected with miR-221 inhibitor was much weaker than that of those transfected with miR-NC (Figures 3(c) and 3(d)). For transwell invasion assays, the number of cells that passed through a Matrigel-coated membrane onto the lower chamber was significantly less in the miR-221 inhibitor transfected cells than in miR-NC transfected cells (Figure 3(e)). The number of invading cells after transfection with miR-NC or miR-221 inhibitor was 82 ± 6 and 47 ± 6, respectively (*P* < 0.05) (Figure 3(f)).

### 3.4. miR-221 Regulates MBD2 Expression by Targeting Its 3′-UTR

**Figure 4:**
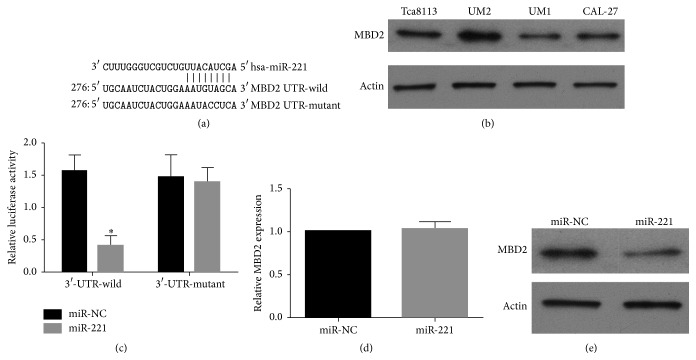
MBD2 is a direct target of miR-221. (a) Predicted duplex formation between the wild-type or mutant MBD2 3′-UTR and miR-221. (b) Western blot showing MBD2 protein expression level in OSCC cell lines. Actin was used as an internal loading control. (c) Luciferase activity of wild-type (3′-UTR-wild) or mutant (3′-UTR-mutant) MBD2 3′-UTR-containing reporters in UM1 cells transfected with miR-221 mimic or miR-NC. (d) qRT-PCR of MBD2 mRNA in UM1 cells transfected with miR-221 mimic or miR-NC. Data were normalized to GAPDH mRNA. Data are expressed as mean ± SD; ^*∗*^ *P* < 0.05. (e) Western blot of MBD2 in UM1 cells transfected with miR-221 mimic or miR-NC. Actin was used as an internal loading control.

To elucidate the underlying mechanism by which miR-221 suppresses migration and invasion of UM1 cells, we explored miR-221 targets using the Targetscan and miRanda bioinformatics algorithms. Our analysis revealed that MBD2 was a potential target of miR-221 based on a putative conserved target sequence at position 291–298 of the MBD2 3′-UTR (Figure 4(a)). To confirm the relationship between miR-221 and MBD2, we first examined the protein levels of MBD2 in the UM1, CAL-27, UM2, and Tca8113 cell lines. Our results revealed lower levels of MBD2 protein in the highly metastatic UM1 and CAL-27 cell lines compared to the less metastatic UM2 and Tca8113 cell lines (Figure 4(b)). To further examine whether miR-221 directly targets MBD2, luciferase reporter vectors containing wild-type or mutant versions of the predicted miR-221 binding sequences in the MBD2 3′-UTR were cotransfected with miR-221 mimic or miR-NC into UM1 cells. Luciferase assays were performed 48 h after transfection. A significant decrease in the luciferase activity of the reporter was observed for the wild-type MBD2 3′-UTR-containing vector compared to miR-NC (Figure 4(c)). This significant decrease in reporter activity was not seen when the reporter was in the vector containing the mutant MBD2 3′-UTR (Figure 4(c)), in spite of the presence of miR-221, indicating that the sequence in the 291–298 bp region of the MBD2 3′-UTR indeed interacts with miR-221 and inhibits the expression of MBD2. We then examined the effects of miR-221 overexpression on MBD2 mRNA and protein levels. Overexpression of miR-221 did not cause degradation of MBD2 mRNA (Figure 4(d)). However, a clear reduction in the level of endogenous MBD2 protein was observed (Figure 4(e)).

### 3.5. MBD2 Is Involved in miR-221 Induced Effects on Migration and Invasion in UM1 Cells

**Figure 5:**
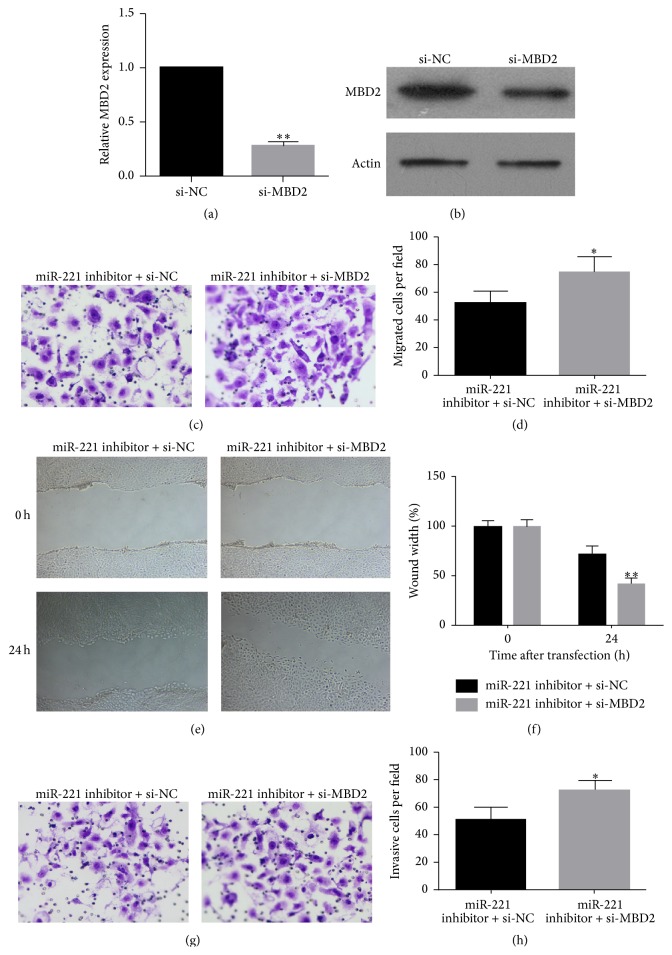
The effect of MBD2 silencing on cell migration and invasion of UM1 cells after miR-221 transfection. (a) MBD2 mRNA expression 48 h after transfection with si-MBD2 or si-NC. (b) Western blot of MBD2 protein expression 48 h after transfection with si-MBD2 or si-NC. (c) Representative images of UM1 cell migration are shown. The migration of UM1 cells was measured using a transwell assay at 48 h after the transfection of miR-221 inhibitor plus si-NC or miR-221 inhibitor mimic plus si-MBD2. (d) The average number of migrating cells per field among the indicated experimental groups is shown. (c) Representative images of UM1 migration cells analyzed by wound healing assays. Images show migration of cells after 0 h and 24 h. (d) Quantification of migrated UM1 cells analyzed by wound healing assays. The migration of UM1 cells transfected with miR-221 inhibitor mimic plus si-NC set to 100%. (g) Representative images of UM1 invasion are shown. The invasion of UM1 cells was measured using a Matrigel invasion assay at 48 h after the transfection of miR-221 inhibitor mimic plus si-NC or miR-221 inhibitor mimic plus si-MBD2. (h) The average number of invading cells per field for the indicated experimental groups is shown. Data are presented as means ± SD. ^*∗*^ *P* < 0.05.

To examine whether miR-221 affects UM1 migration and invasion through MBD2, UM1 cells were transfected with si-MBD2. As shown in Figures 5(a) and 5(b), MBD2 mRNA and protein levels decreased upon transfection with si-MBD2, compared to si-NC. For the transwell migration assay, the number of cells that passed through the membrane onto the lower chamber was significantly higher in the miR-221 inhibitor plus si-MBD2 transfected cells than in miR-221 inhibitor plus si-NC transfected cells (Figure 5(c)). The number of migrating cells after transfection with miR-221 inhibitor plus si-NC or miR-221 inhibitor plus si-MBD2 was 52 ± 9 and 75 ± 10, respectively (*P* < 0.05) (Figure 5(d)). In addition, the wound healing assay showed that the migratory ability of UM1 cells transfected with miR-221 inhibitor plus si-MBD2 was much greater than that of those transfected with miR-221 inhibitor plus si-NC (Figures 5(e) and 5(f)). For the transwell invasion assays, the number of cells that passed through the Matrigel-coated membrane onto the lower chamber was significantly higher in the miR-221 inhibitor plus si-MBD2 transfected cells than in miR-221 inhibitor plus si-NC transfected cells (Figure 5(g)). The number of invading cells after transfection with miR-221 inhibitor plus si-NC or miR-221 inhibitor plus si-MBD2 was 51 ± 10 and 72 ± 7, respectively (*P* < 0.05) (Figure 5(h)). These results suggest that miR-221 affects migration and invasion in UM1 cells through regulation of its target MBD2.

## 4. Discussion

MiRNAs have been shown to play a dual role in tumor invasion and metastasis [19]. On the one hand, miRNAs could promote breast cancer metastasis, specifically miR-10b, as demonstrated by Ma et al. [20]. On the other hand, a set of miRNAs capable of suppressing metastasis in vivo via ectopic restoration was identified, including miR-126 in breast cancer [21]. In addition to various miRNAs playing alternative roles in the same cancer, one miRNA might play a different role in different cancer cells, including miR-221. An increasing number of studies have demonstrated that miR-221 can function as a potential oncogene or an oncosuppressor [11]. One previous study has demonstrated that increased miR-221 expression was associated with OSCC cell growth [15]. However, the effect of miR-221 on OSCC cell migration and invasion is not clear. Therefore, we aimed to investigate the regulatory role of miR-221 on OSCC cell migration and invasion. Our results indicate that miR-221 is highly expressed in highly metastatic OSCC cell lines. Moreover, inhibition of miR-221 suppressed migration and invasion in the highly metastatic OSCC cell line UM1. Similar results were seen in other cancer cells. For example, in prostate cancer, renal cell carcinoma, and gliomas, miR-221 could promote cancer cell migration or invasion [22–24].

miRNAs function by regulating the expression of target genes by either inducing mRNA degradation or inhibiting mRNA translation through imperfect base-pairing with the 3′-UTR of target mRNAs [25–27]. Given the function of miR-221 in regulating cell migration and invasion, genes related to migration and invasion are putative targets. MBD2 is one of the putative targets related to migration and invasion [28]. In this study, MBD2 was identified as a direct target of miR-221 in the OSCC cell line UM1. This result is supported by several findings: (1) a complementary sequence of miR-221 was identified in the 3′-UTR of MBD2 mRNA, suggesting this 3′-UTR interacts with miR-221; (2) overexpression of miR-221 led to a significant reduction in MBD2 protein expression; (3) overexpression of miR-221 suppressed the luciferase reporter activity of a MBD2 3′-UTR-containing vector; (4) this effect was abolished by mutation of the miR-221 binding site in the MBD2 3′-UTR; and (5) MBD2 silencing could reverse the suppressive effect of the miR-221 inhibitor on cell migration and invasion.

MBD2 belongs to a family of MBD domain containing proteins, including MBD1, MBD2, MBD3, MBD4, and MeCP2, which associate with heterochromatin in the nucleus through an interaction with methylated DNA at CpG islands [29]. In gastric cancer, reduced mRNA expression levels of MBD2 were detected [30]. In the present study, reduced MBD2 protein expression was also observed in highly metastatic OSCC cell lines. This expression profile indicates that MBD2 might play a role in regulating migration and invasion. Our results suggest that MBD2 silencing could reverse the suppressive effect of the miR-221 inhibitor on cell migration and invasion. These results suggest that MBD2 might activate genes that suppress migration and invasion in the OSCC cell line UM1. However, previous studies have shown that MBD2 is required for the activation and maintenance of a demethylated state of prometastatic genes in liver, prostate, and breast cancers [31–33]. Therefore, we predict that MBD2 might play different roles through various downstream genes.

In conclusion, we determined that miR-221 is highly expressed in highly metastatic OSCC cell lines, and downregulation of miR-221 inhibits cell migration and invasion partly through targeting MBD2 in the human OSCC cell line UM1.

## Figures and Tables

**Table 1:** Primers for qRT-PCR.

Primer name	Sequence (5′-3′)
miR-miR-221	AGCTACATTGTCTGCTGGGTTTC
miR-miR-221 RT	CTCAACTGGTGTCGTGGAGTCGGCAATTCAGTTGAGGAAACCCA
miR-miR-221 F	ACACTCCAGCTGGGAGCTACATTGTC
U6 F	CTCGCTTCGGCAGCACA
U6 R	AACGCTTCACGAATTTGCGT
Universal R	CTCAACTGGTGTCGTGGA
MBD2 F	AGACCCACAACGAATGAATGAAC
MBD2 R	CTGGACAACTCCTTGAAGACC
GAPDH-F	ACACCCACTCCTCCACCTTT
GAPDH-R	TTACTCCTTGGAGGCCATGT

F: forward primer, R: reverse primer, and RT: reverse transcription primer.

**Table 2:** Primers for luciferase reporter construction.

Primer name	Sequence (5′-3′)
psiCHECK2-XhoI-F	CCGctcgagGAATATGATCAGGTAACTTTCGACCG
psiCHECK2-NotI-R	ATAAGAATgcggccgc ACTCCCTCCCTTCCTTGGTATCAG
psiCHECK2-mut-F	GCCAGGTGCAATCTACTGGAAATACCTCACTTACGTAAAACATTTGTTTCC
psiCHECK2-mut-R	**GGAAACAAATGTTTTACGTAAGTGAGGTATTTCCAGTAGATTGCACCTGGC**

F: forward primer and R: reverse primer.

## References

[1] Yu T., Wu Y., Helman J. I., Wen Y., Wang C., Li L. (2011). CXCR4 promotes oral squamous cell carcinoma migration and invasion through inducing expression of MMP-9 and MMP-13 via the ERK signaling pathway. *Molecular Cancer Research*.

[2] Choi S., Myers J. N. (2008). Molecular pathogenesis of oral squamous cell carcinoma: implications for therapy. *Journal of Dental Research*.

[3] Zini A., Czerninski R., Sgan-Cohen H. D. (2010). Oral cancer over four decades: epidemiology, trends, histology, and survival by anatomical sites. *Journal of Oral Pathology and Medicine*.

[4] de Aguiar Júnior F. C. A., Kowalski L. P., de Almeida O. P. (2007). Clinicopathological and immunohistochemical evaluation of oral squamous cell carcinoma in patients with early local recurrence. *Oral Oncology*.

[5] Ambros V. (2004). The functions of animal microRNAs. *Nature*.

[6] Bartel D. P. (2004). MicroRNAs: genomics, biogenesis, mechanism, and function. *Cell*.

[7] Liz J., Esteller M. (2015). lncRNAs and microRNAs with a role in cancer development. *Biochimica et Biophysica Acta (BBA)—Gene Regulatory Mechanisms*.

[8] Wang W., Zhang E., Lin C. (2015). MicroRNAs in tumor angiogenesis. *Life Sciences*.

[9] Liu Z., Xu Y., Long J., Guo K., Ge C., Du R. (2015). MicroRNA-218 suppresses the proliferation, invasion and promotes apoptosis of pancreatic cancer cells by targeting HMGB1. *Chinese Journal of Cancer Research*.

[10] Tian K., Di R., Wang L. (2015). MicroRNA-23a enhances migration and invasion through PTEN in osteosarcoma. *Cancer Gene Therapy*.

[11] Garofalo M., Quintavalle C., Romano G., Croce C. M., Condorelli G. (2012). miR221/222 in cancer: their role in tumor progression and response to therapy. *Current Molecular Medicine*.

[12] Yang F., Wang W., Zhou C. (2015). miR-221/222 promote human glioma cell invasion and angiogenesis by targeting TIMP2. *Tumor Biology*.

[13] Cai K., Shen F., Cui J. H., Yu Y., Pan H. Q. (2015). Expression of miR-221 in colon cancer correlates with prognosis. *International Journal of Clinical and Experimental Medicine*.

[14] Khella H. W., Butz H., Ding Q. (2015). miR-221/222 are involved in response to sunitinib treatment in metastatic renal cell carcinoma. *Molecular Therapy*.

[15] Yang C.-J., Shen W. G., Liu C.-J. (2011). miR-221 and miR-222 expression increased the growth and tumorigenesis of oral carcinoma cells. *Journal of Oral Pathology and Medicine*.

[16] Nakayama S., Sasaki A., Mese H., Alcalde R. E., Matsumura T. (1998). Establishment of high and low metastasis cell lines derived from a human tongue squamous cell carcinoma. *Invasion and Metastasis*.

[17] Gioanni J., Fischel J.-L., Lambert J.-C. (1988). Two new human tumor cell lines derived from squamous cell carcinomas of the tongue: establishment, characterization and response to cytotoxic treatment. *European Journal of Cancer and Clinical Oncology*.

[18] He R., Xu Q., Zhou X. (1983). The establisment and some bilogical characteristics of a squamous cell line of the human tongue Tca8113. *Tumor*.

[19] Orellana E. A., Kasinski A. L. (2015). MicroRNAs in cancer: a historical perspective on the path from discovery to therapy. *Cancers*.

[20] Ma L., Teruya-Feldstein J., Weinberg R. A. (2007). Tumour invasion and metastasis initiated by microRNA-10b in breast cancer. *Nature*.

[21] Tavazoie S. F., Alarcón C., Oskarsson T. (2008). Endogenous human microRNAs that suppress breast cancer metastasis. *Nature*.

[22] Yang X., Yang Y., Gan R. (2014). Down-regulation of miR-221 and miR-222 restrain prostate cancer cell proliferation and migration that is partly mediated by activation of SIRT1. *PLoS ONE*.

[23] Lu G. J., Dong Y. Q., Zhang Q. M. (2015). miRNA-221 promotes proliferation, migration and invasion by targeting TIMP2 in renal cell carcinoma. *International Journal of Clinical and Experimental Pathology*.

[24] Cai G., Qiao S., Chen K. (2015). Suppression of miR-221 inhibits glioma cells proliferation and invasion via targeting SEMA3B. *Biological Research*.

[25] He L., Hannon G. J. (2004). MicroRNAs: small RNAs with a big role in gene regulation. *Nature Reviews Genetics*.

[26] Valencia-Sanchez M. A., Liu J., Hannon G. J., Parker R. (2006). Control of translation and mRNA degradation by miRNAs and siRNAs. *Genes & Development*.

[27] Winter J., Jung S., Keller S., Gregory R. I., Diederichs S. (2009). Many roads to maturity: microRNA biogenesis pathways and their regulation. *Nature Cell Biology*.

[28] Cheishvili D., Chik F., Li C. C. (2014). Synergistic effects of combined DNA methyltransferase inhibition and MBD2 depletion on breast cancer cells; MBD2 depletion blocks 5-aza-2'-deoxycytidine-triggered invasiveness. *Carcinogenesis*.

[29] Mei L., Xiong W.-C. (2010). FAK interaction with MBD2: a link from cell adhesion to nuclear chromatin remodeling?. *Cell Adhesion and Migration*.

[30] Pontes T. B., Chen E. S., Gigek C. O. (2014). Reduced mRNA expression levels of MBD2 and MBD3 in gastric carcinogenesis. *Tumor Biology*.

[31] Stefanska B., Huang J., Bhattacharyya B. (2011). Definition of the landscape of promoter DNA hypomethylation in liver cancer. *Cancer Research*.

[32] Shukeir N., Pakneshan P., Chen G., Szyf M., Rabbani S. A. (2006). Alteration of the methylation status of tumor-promoting genes decreases prostate cancer cell invasiveness and tumorigenesis *in vitro* and *in vivo*. *Cancer Research*.

[33] Pakneshan P., Têtu B., Rabbani S. A. (2004). Demethylation of urokinase promoter as a prognostic marker in patients with breast carcinoma. *Clinical Cancer Research*.

